# Beyond the clinic: The role of the psychiatrist in practicing pragmatic solidarity

**DOI:** 10.1371/journal.pmen.0000705

**Published:** 2026-08-28

**Authors:** Anne Berhe, Abishek Bala, Tobi Okopie, Jennifer Agwagom, Manal Khan, Suzan J. Song

**Affiliations:** 1 Department of Psychiatry, Columbia University, New York, New York, United States of America; 2 Department of Child & Adolescent Psychiatry, Children’s Hospital of Eastern Ontario, University of Ottawa, Ottawa, Ontario, Canada; 3 Department of Psychiatry, Boston Children’s Hospital, Boston, Massachusetts, United States of America; 4 Geisinger Commonwealth School of Medicine, Scranton, Pennsylvania, United States of America; 5 David Geffen School of Medicine, University of California Los Angeles, Los Angeles, California, United States of America; 6 Department of Psychiatry and Behavioral Sciences, George Washington University School of Medicine and Health Sciences, Washington, District of Columbia, United States of America; PLOS: Public Library of Science, UNITED KINGDOM OF GREAT BRITAIN AND NORTHERN IRELAND

## Abstract

Psychiatrists are trained to evaluate biological, psychological, and social dimensions of illness simultaneously, yet the dominant frameworks governing psychiatric practice remain oriented toward individual symptom management rather than the structural conditions that produce these symptoms. This essay argues that psychiatrists working in global mental health contexts carry an ethical obligation that extends beyond diagnosis and treatment: to practice what Paul Farmer termed pragmatic solidarity, the practice of standing alongside communities to address the upstream drivers of mental distress through structural advocacy, community-led care, and institutional accountability. This essay draws on evidence from three intersecting domains. First, it examines the well-documented global mental health burden, including treatment gaps and the disproportionate impact of conflict, displacement, and structural inequity on psychological wellbeing. Second, it argues that effective global mental health work requires horizontal partnerships, epistemic justice, and the centering of community-defined priorities over externally imposed frameworks. Third, it illustrates what pragmatic solidarity looks like in individual, institutional and policy contexts. Importantly, this essay is a call to action, identifying concrete entry points through which psychiatrists can move from recognition of structural drivers to active participation in dismantling them, including through material redistribution, community partnership, participatory research, and policy engagement. Pragmatic solidarity offers psychiatrists not just a theoretical framework, but a tangible, enactable way of improving global mental health.

## Introduction

Psychiatrists exist in a role in which the physical, social, and structural realities of their patients are deeply relevant to providing care. When considering psychiatrists who work in the setting of global mental health, these varied environments encompass a distinct array of adverse events that impact psychological wellbeing. The impact of adverse events occurring on a global scale is especially pronounced in an era marked by escalating conflict, widening socioeconomic inequities, and mass displacement, with disproportionate burden falling on those from low- and middle-income countries (LMICs). Just as advocacy for the drivers of poor mental health is integral to local psychiatric practice, psychiatrists working in global contexts should likewise consider their ethical obligations to advocacy at a broader level.

A significant portion of global mental health scholarship reinforces Western interpretations, and as psychiatrists with varied personal and professional experiences, the authors of this essay hope to decenter the colonial presence over the conversation. Collectively, we include individuals of various African, East Asian, and South Asian backgrounds, with varied migration histories, including first- and second-generation American immigrants. While all authors are currently affiliated with institutions in North America, members of the team have lived, trained, and worked across multiple low-, middle-, and high-income countries. We recognize that our institutional affiliations confer privilege and influence our perspectives, and have sought to approach this work with reflexivity, valuing diverse viewpoints within the author team while acknowledging the limitations inherent in our positionalities.

A few terms are important for understanding the state of global mental health. Global psychiatry refers to the field of medicine focused on reducing the burden of psychiatric disorders across the globe, often with an emphasis on equity through research, capacity building, and education. Decolonization refers to the process of dismantling imperial structures; and in global health, decolonization refers more specifically to dismantling Western-centric approaches in favor of indigenous voices, structures, and epistemologies [1–3]. Decolonization when reformulated by indigenous peoples pushes back on hegemonic agendas, or the interests of dominant groups such as Western governments, elite university systems, and community level aristocrats, that exercise authority over historically excluded/minimized populations [4]. The Global South refers broadly to countries that have experienced histories of colonization and continue to occupy relatively marginalized positions within the global political and economic order, whereas Global North refers to countries that hold greater power due to histories of colonialism, resource extraction and unequal global exchange. Liberation psychology, founded by social psychologist Ignacio Martín-Baró, is a field adjacent to global psychiatry that focuses on the psychological impact of systemic inequality on marginalized people [5]. It prioritizes the experiences of those in marginalized groups by explicitly naming oppression as a driver of psychological distress and linking liberation work to the practice of mental health care [6]. Pragmatic solidarity is a social justice framework coined by the late Paul Farmer that can be applied to global psychiatry; it emphasizes practical, immediate support to alleviate suffering while addressing the root causes of illness [7].

This essay is a call for psychiatrists to apply pragmatic solidarity in their global mental health work. When social arrangements systemically produce trauma, hopelessness, and despair, we must ask how we can advocate to disrupt those arrangements. This view situates psychiatric practice not only to alleviate symptoms, but also as a means to eliminate their upstream drivers. As Farmer described, pragmatic solidarity goes beyond acknowledgment: *“suffering with’ is really not very pragmatic unless it is linked to reducing suffering… It is not just about acknowledging the suffering of others. It is also about asking the question, ‘how much of this suffering is premature or even unnecessary and what might we do collectively to lessen it?’”* [8]. Empathy becomes pragmatic solidarity when it is accompanied by action to reduce suffering [9].

Pragmatic solidarity has notably influenced global health initiatives in infectious diseases, perhaps most notably through the DOTS-Plus strategy for treating multidrug-resistant tuberculosis (MDR-TB) [10]. Partners in Health provided costly MDR-TB regimens in low-income countries despite WHO recommendations against such distribution, demonstrating that MDR-TB can be successfully treated in resource-poor settings. Alongside pharmaceutical TB treatment, Partners in Health also provided patients with food packages to improve nutrition, support from community health workers, and representation through advocacy to the United States Senate on how increased TB deaths were influenced by policy adversely impacting countries such as Haiti [11]. This approach recognizes socioeconomic barriers to care and pushes back on hegemonic agendas to tangibly allocate resources for populations rendered vulnerable by structural and political inequities. Pragmatic solidarity within psychiatry demands the same orientation: *recognizing* that many forms of distress are responses to violence, *confronting* the social, political, and historical forces that shape mental health, and *intervening* on the structural conditions that give rise to psychological suffering, deprivation, and exclusion [12].

## Engaging with social and material realities

When practicing at the full scope of their training, psychiatrists bring a biopsychosocial lens to clinical care. Distinct from the biomedical model, this framework highlights the importance of contextual realities. Physical and mental health are shaped by social drivers, including economic stability, education access, healthcare quality, neighborhood environment, and social and community context. On the global scale, these social drivers are exacerbated due to oppressive systems rooted in capitalism, environmental exploitation, and anti-people political agendas to name a few. The landmark Adverse Childhood Experiences (ACE) study established the long-term deleterious effects of toxic stress on physical and mental health, substance use, and social wellbeing, providing foundational evidence that social conditions are not peripheral to clinical care but central to it [13].

Acknowledging social drivers allows physicians, including psychiatrists, to understand patient suffering more accurately and respond more effectively. Liberation psychology shifts the lens of the clinician from focusing on individual deficits to addressing the intersecting systems of oppression (e.g., racism, homophobia, economic inequality) that drive illness [5]. Lack of sleep due to housing insecurity is distinct from insomnia rooted in a mood disorder; weight loss from food poverty differs from weight loss driven by an eating disorder; trauma from ongoing war is distinct from PTSD. When social drivers are not considered, clinicians risk misdiagnosis, harmful interventions, and a clinical relationship that fails the patient. These risks fall disproportionately on minoritized and marginalized populations.

Indigenous populations exemplify how social drivers shape risk as many Indigenous groups experience disproportionately high rates of suicide compared to non-Indigenous populations, as evidenced by a systematic review of 99 studies across 30 countries and territories [14]. The compounding impact of colonialism, intergenerational trauma, poverty, and cultural dislocation undoubtedly shape rates of depression and suicidality in these communities [15]. Ansloos contextualizes current interventions on Indigenous suicidality as solely focused on “risk assessment, management, detection, and treatment of mental illness” while negating the multidimensional being of the Indigenous person [16].

Overdiagnosis of psychotic disorders in Black patients, despite no evidence for differences in prevalence, is well documented and leads to unnecessary exposure to antipsychotic medications, higher doses, and long-acting injectable formulations, increasing the risk of adverse effects such as tardive dyskinesia and further stigmatization [17,18]. Conversely, Black and Latino children are less likely than White children to be diagnosed with ADHD and less likely to receive guideline-driven care, reflecting a parallel pattern of underdiagnosis and undertreatment [19–21].

On a structural level, underfunding of health systems serving marginalized populations is a significant driver of these disparities. Treatment gaps in LMICs are substantially higher than in other regions, with over 75% of people with a diagnosable mental illness in LMICs not receiving treatment [22]. While high-income countries allocate 6 to 11 percent of health budgets to mental health, LMICs average below 1 percent. Chronic underfunding perpetuates the social conception that mental health is not a priority while simultaneously producing treatment delays that increase the severity and complexity of psychiatric illness. Unhoused individuals, socioeconomically disadvantaged individuals, immigrants, and those with limited English language proficiency each face compounding risk for negative mental health outcomes [23–27]. Together, this evidence makes clear that psychiatrists cannot fulfill their clinical obligations without engaging in the social and material conditions that shape their patients’ lives.

## Pragmatic solidarity in global mental health

Psychiatric practice takes place within a socioecological continuum in which individuals are shaped by family, community, and society, and these systems are shaped in turn by the collective experience of individuals [28]. This bidirectionality allows psychiatrists to integrate clinical insight with broader sociocultural environments. When psychiatrists work transnationally, they become involved with diverse networks spanning academic institutions, governmental agencies, international organizations, and community partners. By serving as the connection between resource-generating and resource-receiving institutions, psychiatrists facilitate the movement of knowledge, funding, training, and influence across settings and cultures, creating both opportunity and ethical obligation.

Pragmatic solidarity requires psychiatrists to understand how resources move through institutional systems and to ensure that the benefits of global mental health initiatives reach the communities for whom they are intended [29]. This demands a broadened professional role, one oriented not toward institutional agendas but toward strengthening communities and promoting social structures rooted in justice and equality. Achieving this shift requires horizontal partnerships, mutual learning, and community-led processes that counteract forces prioritizing institutional growth over community wellbeing [30]. Psychiatrists are therefore called to act as stewards who direct resources toward equity, community priorities, and sustainable capacity building [31,32].

The social contexts shaping mental health are themselves products of power and inequity, including colonialism, racism, and class stratification [33]. This reframes the clinical gaze: the treatment of symptoms becomes inseparable from the structural conditions that give rise to them, and the psychiatrist’s role expands beyond diagnosis and management to include partnership in navigating, and when possible challenging, those conditions. Research in global mental health stresses that communities must define their own priorities and models of wellbeing rather than

having external frameworks imposed upon them [34]. Achieving this requires extending the collaborative principles that guide clinical practice into the global arena, creating environments in which diverse perspectives are heard, power imbalances are actively countered, and historically excluded voices are elevated. This reflects the principle of epistemic justice, which recognizes communities as legitimate producers of knowledge and ensures their expertise shapes the development of mental health initiatives [30].

These commitments are rooted in the clinical ethics of psychiatry itself. Clinicians regularly work with individuals whose agency may be constrained by illness, and they are trained to protect patients’ perspectives and rights under those conditions. The same principles apply globally. Psychiatrists must avoid imposing hegemonic frameworks and instead create conditions in which communities can articulate their needs, determine their priorities, and build structures reflecting their own values. Participatory approaches such as Community-Based Participatory Research (CBPR) reorient care by incorporating patient agency within care delivery, while frameworks such as the NIMH Research Domain Criteria (RDoC) sociality domain allow clinicians to understand social functioning as a continuum shaped by environmental context rather than fixed diagnostic categories [32]. Together, these tools support pragmatic solidarity within routine psychiatric practice, strengthening engagement, empowering patients, and advancing the structural competence that effective global mental health work demands.

## Systems collaboration

Mental health outcomes are shaped by systems that extend well beyond the clinical encounter. Clinical interventions may fall short when the factors influencing access and engagement are neglected [35]. Understanding mental health as a product of interacting systems enables psychiatrists to address the needs of individuals whose distress is shaped by their broader environment.

Systems science offers models that inform approaches to mental health care delivery. A health systems perspective examines care through domains such as governance and workforce development, highlighting the importance of coordination across management and service sectors [36]. Because many determinants of mental health arise from social and economic conditions, social protection programs and community-based supports play a central role in mitigating risk [31]. Social protection in this context refers to safety net initiatives, including food security programs, that reduce the effects of poverty and social exclusion.

Sustained progress depends on consistent communication and ongoing information sharing across systems, allowing interventions to remain responsive to changing community needs. Within this framework, psychiatrists can engage in systems collaboration by developing sustained relationships with community partners who understand the structural challenges patients face.

## Policy translation and agenda-setting frameworks

Systems collaboration helps psychiatrists recognize where structural barriers arise, but meaningful improvement often depends on the laws and regulations that govern mental health care. Policy translation reflects the understanding that supporting patients requires addressing the conditions that shape community resources. When psychiatrists bring both clinical experience and community perspectives into policy discussions, they help ensure that mental health remains visible within government agendas.

The Evidence to Agenda-Setting (EVITA) framework provides a structured way to understand how evidence can influence policy, highlighting the importance of building coalitions and forming relationships with groups who understand community needs [37]. EVITA also demonstrates that policy change is often gradual and requires consistent effort rather than a single intervention. Global mental health initiatives such as WHO’s Mental Health Gap Action Programme (mhGAP) demonstrates how guidelines can support national leaders in expanding access to care by training general health workers and strengthening local services [38]. The Movement for Global Mental Health focuses on human rights and community leadership in shaping national priorities, placing lived experience at the center of policy design. Both initiatives reflect the principle that mental health systems improve when governments invest in capacity building and when community voices influence how resources are used. The need for capacity building has become increasingly urgent in a geopolitical climate that has highlighted the fragility of partnerships between the Global North and Global South, exemplified by the United States’ recent withdrawal from the World Health Organization and dismantling of USAID. While these partnerships are on one hand a means of exerting soft power, their sudden reversal has also dramatically destabilized programs and communities, reinforcing the importance of having global mental health policy that minimizes reliance on a dominant entity [39].

## Ethical issues in structural engagement

Ethical clarity is essential when psychiatrists enter spaces beyond the clinic to address structural conditions shaping mental health. As partnerships expand and institutional oversight shrinks, the risk of acting without accountability increases. Well-intentioned involvement can produce harm when professionals impose solutions that do not reflect community priorities or histories [40]. The distinction between solidarity and saviorism is central to structural work. Solidarity involves shared purpose, co-learning, and responsiveness to local knowledge; saviorism arises when clinicians assume authority over problems they do not fully understand. Ethical structural engagement also requires challenging the notion of clinical neutrality, which can function to uphold existing systems of inequity [5]. Psychiatrists should instead position themselves within institutions of power to act as structural advocates, promoting interventions that actively diminish structural violence.

Humility and reciprocity guide sustainable partnerships. Reciprocity involves creating relationships where knowledge and benefits move in both directions, while humility acts as a protective force against reproducing colonial patterns of authority within mental health practice [41]. This approach supports the decolonization of global mental health, shifting power so that post-colonial nations and local communities have primary agency over the research and policy agendas that affect them. Long-term partnerships, rather than short-term initiatives, help build trust and support interventions that remain meaningful even after external collaborators step back.

Self-reflection on bias is another ethical cornerstone of structural engagement. Clinicians must examine their own racial, cultural, and professional positionalities to avoid perpetuating structural harm [42]. Structural competence requires more than awareness of social determinants; it includes recognizing how one’s own training and identity shape interactions within clinical and community settings. This introspective work creates space for more honest, accountable collaboration and reduces the likelihood of reinforcing inequities.

Ethical engagement also requires attention to workforce diversity and the empowerment of marginalized voices. In a global context, diversity must be defined beyond Western-centric racial categories to include intersectionality and representation across linguistic, class, and cultural lines relevant to the local environment. A diverse mental health workforce improves cultural responsiveness, reduces structural blind spots, and strengthens community trust [43]. Participatory approaches that center marginalized leadership help ensure that structural interventions reflect lived realities rather than institutional assumptions. Ethical practice in this context means sharing power, supporting community-driven priorities, and strengthening pathways for underrepresented groups to shape the future of mental health systems.

## Call to action

Psychiatrists are uniquely positioned to address not only the psychological dimensions of suffering but also its structural foundations. Trained to evaluate biological, social, and psychological forces simultaneously, psychiatrists are equipped to identify the external conditions that shape a patient’s presentation. This capacity must extend beyond the individual clinical encounter. A critical gap in current practice is the failure to move from recognition to prevention: from addressing symptoms to dismantling the upstream conditions that produce them. Physicians are more than healers; they are advocates whose professional obligation includes bridging the space between individual care and collective wellbeing.

Pragmatic solidarity offers a framework for that bridging work. It allows psychiatrists to contextualize the full human experience and address the structural drivers of distress rather than cycling through symptomatic treatment without attending to cause. Some practical entry points include: writing policy statements and engaging with local and national government; using professional authority to publicly oppose violence and human rights violations; recognizing social drivers of health and addressing gaps in relevant community resources; partnering with local organizations to better understand structural context; and contributing to or conducting participatory research. Psychiatrists with institutional power can further leverage their positions to provide material resources through mechanisms which include: redirecting grant funding to locally-led initiatives; sharing authorship, leadership, and decision-making authority; supporting reparative funding models; reimbursing social interventions; and supporting medical training for international students whose communities bear disproportionate burdens of psychiatric illness.

The world exists on a scale that demands more than individualized care. Systemic injustices that perpetuate cycles of poverty and structural exclusion will not yield to passive observation or professional neutrality [5]. Progress does not mean the advancement of any single nation’s ideals; it means embracing a shared humanity and investing in the knowledge, capacity, and self-determination of communities everywhere. Psychiatrists who take pragmatic solidarity seriously are not overstepping their role. They are fulfilling it.
